# Protective role of CXCR7 activation in neonatal hyperoxia-induced systemic vascular remodeling and cardiovascular dysfunction in juvenile rats

**DOI:** 10.1038/s41598-023-46422-3

**Published:** 2023-11-09

**Authors:** Merline Benny, Mayank Sharma, Shathiyah Kulandavelu, PingPing Chen, Runxia Tian, Sydne Ballengee, Jiang Huang, Amanda F. Levine, Matteo Claure, Augusto F. Schmidt, Roberto I. Vazquez-Padron, Claudia O. Rodrigues, Shu Wu, Omaida C. Velazquez, Karen C. Young

**Affiliations:** 1 Department of Pediatrics, University of Miami Miller School of Medicine, 1580 NW 10Th Avenue, RM-344, Miami, FL 33136 USA; 2 Batchelor Children’s Research Institute, University of Miami Miller School of Medicine, Miami, FL USA; 3 The Interdisciplinary Stem Cell Institute, University of Miami Miller School of Medicine, Miami, FL USA; 4 Department of Surgery, University of Miami Miller School of Medicine, Miami, FL USA; 5 Department of Molecular and Cellular Pharmacology, University of Miami Miller School of Medicine, Miami, FL USA

**Keywords:** Heart failure, Arterial stiffening

## Abstract

Neonatal hyperoxia induces long-term systemic vascular stiffness and cardiovascular remodeling, but the mechanisms are unclear. Chemokine receptor 7 (CXCR7) represents a key regulator of vascular homeostasis and repair by modulating TGF-β1 signaling. This study investigated whether pharmacological CXCR7 agonism prevents neonatal hyperoxia-induced systemic vascular stiffness and cardiac dysfunction in juvenile rats. Newborn Sprague Dawley rat pups assigned to room air or hyperoxia (85% oxygen), received CXCR7 agonist, TC14012 or placebo for 3 weeks. These rat pups were maintained in room air until 6 weeks when aortic pulse wave velocity doppler, cardiac echocardiography, aortic and left ventricular (LV) fibrosis were assessed. Neonatal hyperoxia induced systemic vascular stiffness and cardiac dysfunction in 6-week-old rats. This was associated with decreased aortic and LV CXCR7 expression. Early treatment with TC14012, partially protected against neonatal hyperoxia-induced systemic vascular stiffness and improved LV dysfunction and fibrosis in juvenile rats by decreasing TGF-β1 expression. In vitro, hyperoxia-exposed human umbilical arterial endothelial cells and coronary artery endothelial cells had increased TGF-β1 levels. However, treatment with TC14012 significantly reduced the TGF-β1 levels. These results suggest that dysregulation of endothelial CXCR7 signaling may contribute to neonatal hyperoxia-induced systemic vascular stiffness and cardiac dysfunction.

## Introduction

Prematurity exposes the vulnerable vascular tree and preterm heart to hostile intrauterine and extrauterine conditions, leading to persistent alterations in cardiovascular function even into adulthood^1,2^. As preterm survival rate has increased, so has the incidence of these morbidities in the first adult generation surviving extreme preterm birth^3–6^. Adults born preterm have an increased risk of hypertension, atherosclerosis, ischemic heart disease, altered cardiac remodeling and early heart failure^7–11^. Understanding the molecular underpinnings for these vascular perturbations and cardiovascular dysfunction is critical to developing effective therapies and improving long-term outcomes.

It has long been demonstrated that neonatal hyperoxia induces oxidative stress and contributes to bronchopulmonary dysplasia and retinopathy of prematurity^12,13^. Previous studies have shown that neonatal hyperoxia exposure induces systemic vascular stiffness, aortic fibrosis and cardiac dysfunction in juvenile rats^14–16^. Endothelial injury and excessive fibrosis are key contributors to this systemic vascular dysfunction but the molecular underpinnings linking oxygen supplementation in early neonatal life and adult vascular dysfunction are essentially unknown.

Chemokine receptors and their ligands play significant roles in vascular development and remodeling^17,18^. Genome wide association studies have identified the genetic locus of CXCL12, which encodes C-X-C motif chemokine 12, also known as stromal cell-derived factor (SDF-1) to be associated with increased risk of heart disease^19^. Chemokine receptor 7 (CXCR7) or atypical chemokine receptor 3 (ACKR3) is a transmembrane spanning receptor of the ligand SDF-1, a cytokine known to play a key role in organ development and repair after injury. Downstream signaling of SDF-1 following binding to its receptors, CXCR7 and chemokine receptor 4 (CXCR4), modulates vascular homeostasis and repair^20^. CXCR7 functions as a decoy receptor for SDF-1, binding it with tenfold higher affinity than CXCR4^21^. CXCR7 impacts angiogenesis by regulating endothelial cell migration and tube formation in vitro^22^. Complete CXCR7 deletion disrupts vascular formation suggesting that CXCR7 is involved at multiple points in cardiovascular development^23^. SDF-1/CXCR7 axis thus plays a pivotal part in endothelial biology and promotes developmental and pathological angiogenesis^24,25^.

Emerging evidence also demonstrates that CXCR7 is a novel regulator of endothelial repair and fibrosis at sites of stress or injury in the endothelium^24,26–28^. Endothelial-specific deletion of CXCR7 produces mesenchymal cell proliferation in cardiac valves, impairs vascular homeostasis, and potentiates cardiac fibrosis^26,29^. Moreover, CXCR7 agonism pharmacologically or via gene delivery decreases post-infarct fibrosis and remodeling^26^. In light of this intricate interplay between SDF-1, CXCR7, and CXCR4, we aimed to dissect the role of CXCR7 in neonatal hyperoxia-induced systemic vascular stiffness and cardiovascular dysfunction. Here we investigated whether neonatal hyperoxia-exposure alters aortic SDF-1/CXCR7 axis and whether pharmacological CXCR7 agonism prevents neonatal hyperoxia-induced systemic vascular stiffness and cardiovascular dysfunction in juvenile rats. Our study has important implications as currently there are no therapeutic strategies to prevent the long-term cardiovascular consequences of preterm birth.

## Results

### Neonatal hyperoxia alters CXCR7 expression in the aorta and left ventricle (LV) of juvenile rats

**Figure 1:**
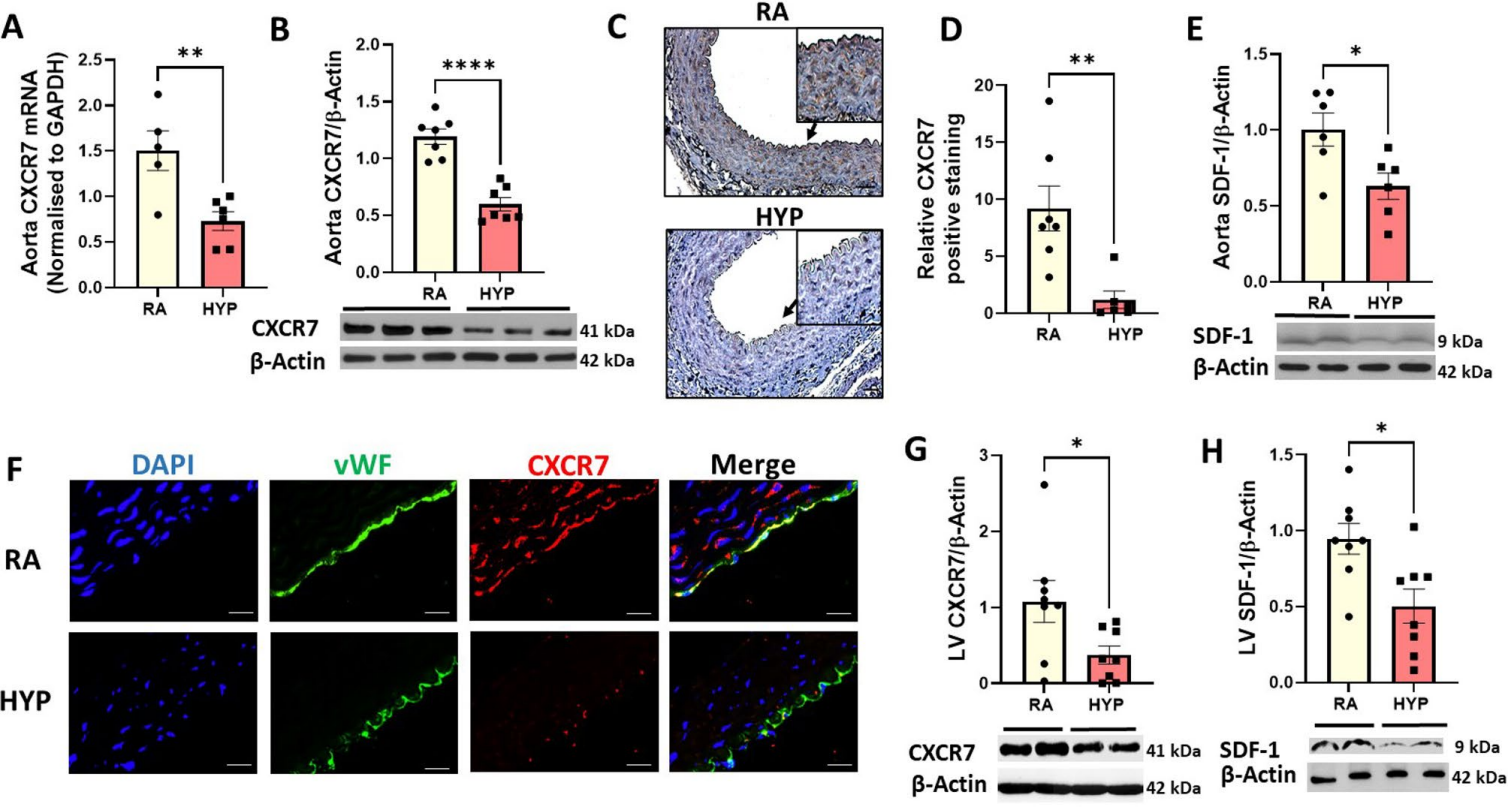
Neonatal hyperoxia exposure decreases aortic and left ventricular CXCR7 and SDF-1 expression in juvenile rats. Decreased aortic (**A**) CXCR7 mRNA (**B**) CXCR7 protein expression (**C**) Representative images of aorta CXCR7 immunostaining (brown staining) and corresponding (**D**) CXCR7 quantification using image J showing decreased CXCR7 in aortas of 6-week-old rats exposed to neonatal hyperoxia (scale bar 50 µm). An inset at 2 × focal enlargement of the areas indicated by the arrows is also given. (**E**) SDF-1 protein expression in 6-week-old rats exposed to neonatal hyperoxia. (**F**) Double immunofluorescent staining with CXCR7 (red) and von Willebrand factor (vWF; green) antibodies, showing decreased endothelial CXCR7 (yellow) in the aorta of hyperoxia-exposed rats. DAPI stains nuclei blue. Decreased left ventricular (**G**) CXCR7 protein expression and (**H**) SDF-1 protein expression in 6-week-old rats exposed to neonatal hyperoxia. Representative Western blot is shown in the lower panel. CXCR7 and SDF-1 expression was normalized to β-Actin, n = 5–8/group, data are mean ± SEM, Student’s unpaired t-test. *p < 0.05, **p < 0.01, ****p < 0.0001; RA = room air; HYP = hyperoxia.

CXCR7 is a key regulator of vascular homeostasis^26,30^. Exposure to neonatal hyperoxia was associated with more than a twofold decrease in CXCR7 gene (*p* value = 0.007) and CXCR7 protein (*p* value < 0.0001) expression in aortas of juvenile rats (Fig. 1A,B). Immunostaining to determine the spatial expression of CXCR7 in aorta demonstrated that CXCR7 was expressed in the tunica intima and tunica media of the aorta (Fig. 1C) and revealed a decrease in CXCR7 expression in the hyperoxia-exposed aortas (Fig. 1D, p value = 0.004). This was accompanied by a 1.6-fold decrease in protein expression of SDF-1 in the aorta (Fig. 1E, p value = 0.02). Moreover, double immunofluorescence staining of aortic sections with vWF (green), a marker of endothelial cells, and CXCR7 (red) showed that CXCR7 is expressed in the endothelial cells (Fig. 1F). Western blot analysis also demonstrated that, when compared to room air-exposed rats, the hyperoxia-exposed rats had a threefold and 1.8-fold decrease in LV CXCR7 (Fig. 1G, p value = 0.03) and SDF-1 expression respectively (Fig. 1H, p value = 0.01).

### Effect of CXCR7 agonism on neonatal hyperoxia-induced systemic vascular stiffness and cardiovascular dysfunction

**Figure 2:**
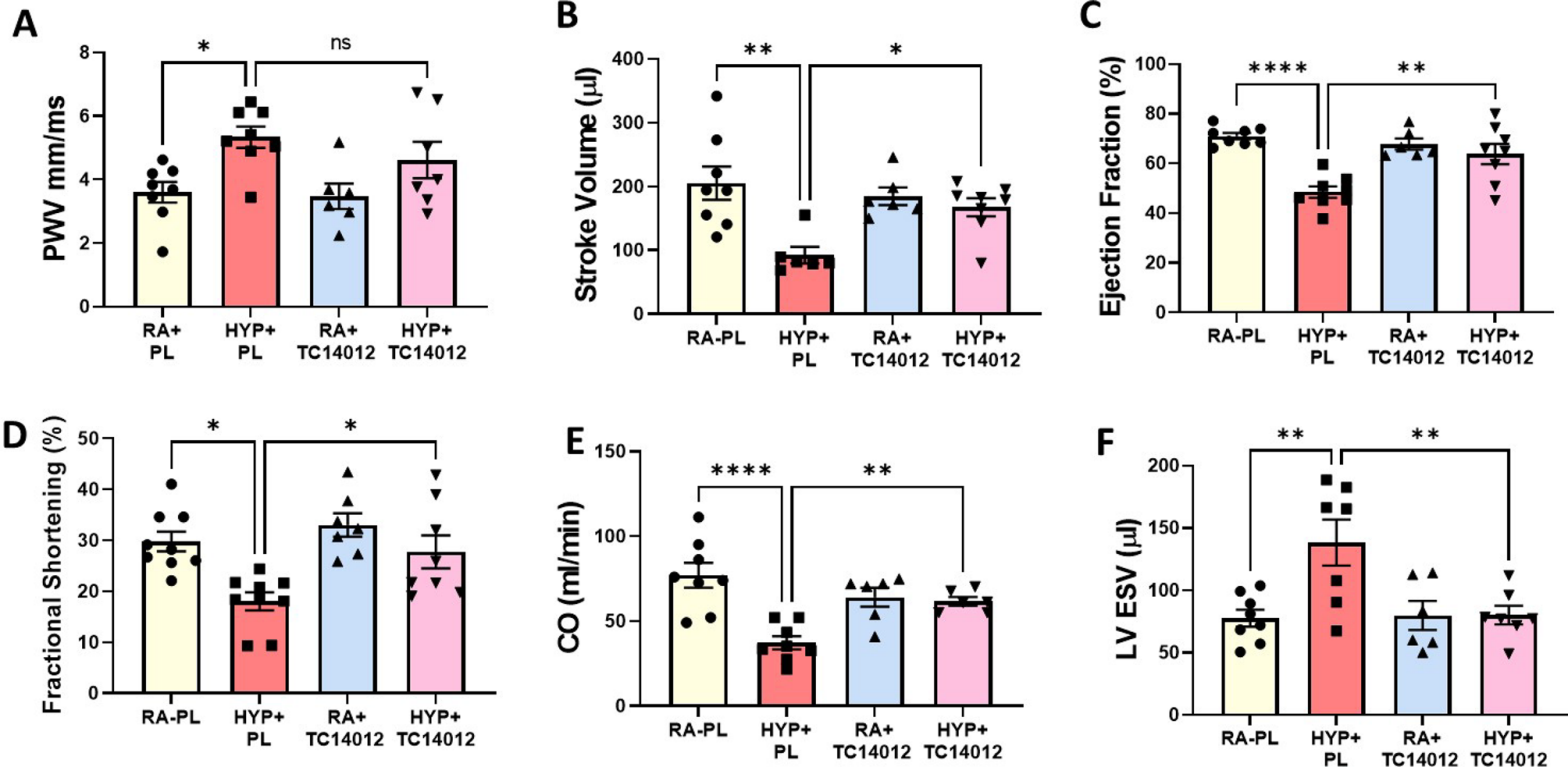
Effects of CXCR7 activation on neonatal hyperoxia induced systemic vascular stiffness and cardiac function in juvenile rats. (**A**) Doppler ultrasound shows that neonatal hyperoxia exposure increased vascular stiffness in juvenile rats, but there was no difference in PWV between the room air treated and hyperoxia treated TC14012 groups. Echocardiography shows that TC14012 administration preserved long-term LV function parameters (**B**) Stroke Volume (**C**) Ejection Fraction (**D**) Fractional Shortening (**E**) Cardiac Output (**F**) Left ventricular End Systolic Volume in the hyperoxia-exposed rats. n = 6–9/group, data are mean ± SEM, Two-way ANOVA with Tukey’s multiple comparisons test. *p < 0.05, **p < 0.01, ****p < 0.0001; RA + PL = room air-exposed placebo treated versus HYP + PL = hyperoxia-exposed placebo treated versus RA + TC14012 = room air treated with TC14012 versus HYP + TC14012 = hyperoxia-exposed treated with TC 14012.

Pulse wave velocity (PWV), an indirect measure of the vascular stiffness of a vessel, has emerged as a powerful tool for the diagnosis of cardiovascular disease and mortality^31,32^. To ascertain whether pharmacological CXCR7 activation protects against neonatal hyperoxia-induced systemic vascular stiffness and cardiovascular dysfunction, we performed PWV and echocardiography. There was no significant difference in PWV and cardiac function between normoxia-exposed placebo (PL) and normoxia-exposed TC14012 treated rats. Whereas hyperoxia-exposed placebo (PL) treated rats had increased aortic stiffness as evidenced by increased PWV, the administration of a specific CXCR7 agonist, TC14012 showed a trend to decreasing hyperoxia-induced aortic stiffness to room air levels but this was not statistically significant (Fig. 2A). Given the intricate relationship between systemic vascular stiffness and cardiac function, we next assessed cardiac function in these rats. Interestingly, cardiac echocardiography demonstrated that hyperoxia PL-treated rats had decreased LV function parameters such as stroke volume, ejection fraction, fractional shortening, cardiac output and increased LV end systolic volume. Administration of TC14012 significantly improved these parameters of LV dysfunction seen in hyperoxia-exposed rats (Fig. 2B–F).

### CXCR7 agonism decreases aortic and LV pro-fibrotic markers in juvenile rats

**Figure 3:**
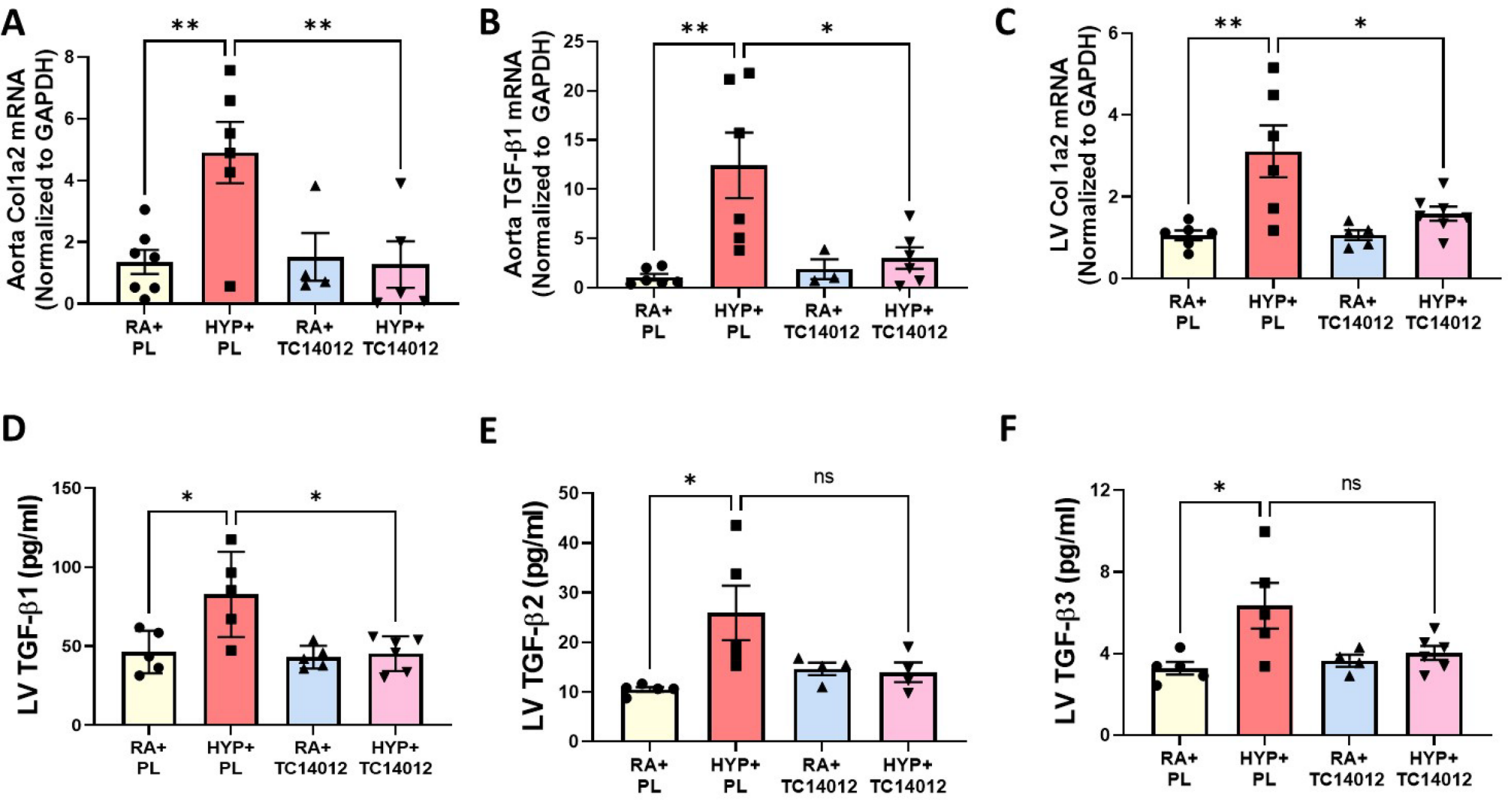
CXCR7 activation attenuates markers of aortic and cardiac fibrosis in juvenile rats. Early supplementation of TC14012 reduced the gene expression of aortic **(A)** collagen 1a2 (Col 1a2) (**B**) TFG-β1 and (**C**) LV Col 1a2 mRNA levels in neonatal hyperoxia-exposed juvenile rats. Multiplex protein analysis of LV homogenate shows (**D**) reduced TFG-β1, (**E**) TFG-β2 and (**F**) TFG-β3 in the hyperoxia-TC1402 treated group. n = 6–7/group, data are mean ± SEM, Two-way ANOVA with Tukey’s multiple comparisons test. *p < 0.05, **p < 0.01, ***p < 0.001; RA + PL = room air-exposed placebo treated versus HYP + PL = hyperoxia-exposed placebo treated versus RA + TC14012 = room air treated with TC14012 versus HYP + TC14012 = hyperoxia-exposed treated with TC14012.

CXCR7 plays a critical role in modulating fibrosis^27^, thus, we next assessed whether CXCR7 activation would alter the expression of pro-fibrotic markers in the aorta and LV of neonatal hyperoxia-exposed rats. As expected, hyperoxia PL-treated rats had increased aortic collagen 1a2 (Col 1a2) (Fig. 3A) and TGF-β1 (Fig. 3B) mRNA levels. These levels were significantly decreased in hyperoxia-exposed rats treated with TC14012. Similarly, hyperoxia PL-treated rats had increased LV Col 1a2 gene expression (Fig. 3C) and TGF-β1-3 protein concentrations (Fig. 3D–F). Administration of TC14012 in hyperoxia-exposed rats decreased LV Col 1a2 mRNA levels and TGF-β1 protein concentration (Fig. 3C–F). CXCR7 agonism did not significantly alter aortic and profibrotic markers in normoxia-exposed rats. This suggests that CXCR7 agonism may improve neonatal hyperoxia-induced cardiovascular fibrosis by decreasing TGF-β1 expression.

### CXCR7 agonism attenuates hyperoxia-induced systemic vascular endothelial cell injury

**Figure 4:**
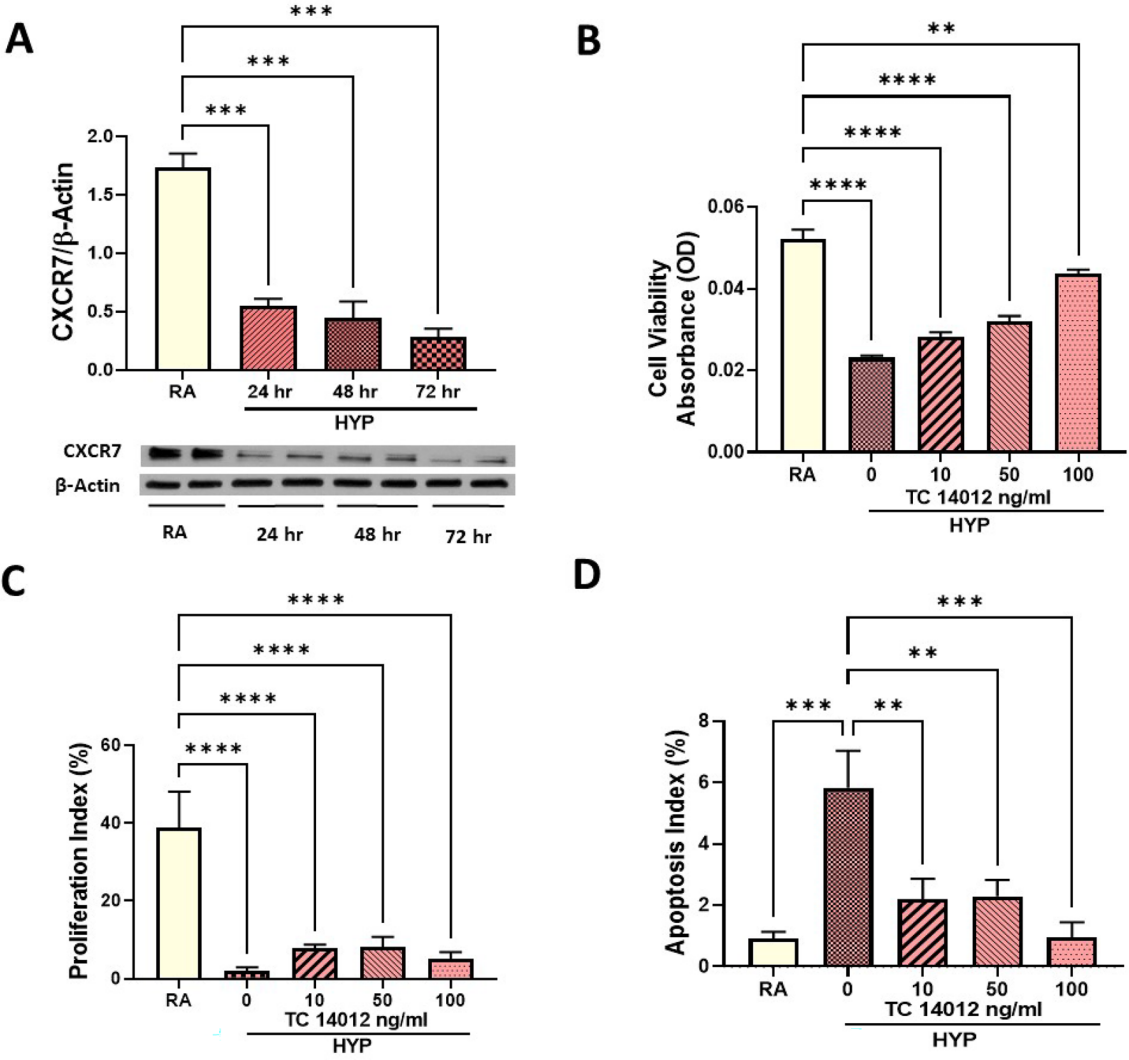
CXCR7 agonism increases cell viability, proliferation and survival in hyperoxia-exposed systemic vascular endothelial cells. (**A**) Western blot analysis showing decreased CXCR7 expression in hyperoxia-exposed human umbilical artery endothelial cells (HUAECs) at 24, 48 and 72 h. Treatment of hyperoxia- exposed HUAECs with TC14102, a CXCR7 agonist increases (**B**) cell viability in MTT assay, (**C**) cell proliferation index (percentage of Ki67^pos^nuclei/total nuclei) and (**D**) cell survival (percentage apoptotic nuclei/total nuclei). Data are mean ± SEM, Two-way ANOVA with Tukey’s multiple comparisons test. **p < 0.01, ***p < 0.001, ****p < 0.0001; RA = normoxia versus HYP = hyperoxia-exposed versus HYP + TC14012 = hyperoxia-exposed treated with TC14012; all experiments were performed in triplicate.

To study the role of endothelial CXCR7 in neonatal hyperoxia-induced systemic vascular dysfunction, we used human umbilical arterial endothelial cells (HUAECs), as the aorta is a direct extension of the umbilical artery in neonates. Exposure to hyperoxia for 24, 48 and 72 h (hr) significantly decreased CXCR7 expression in the HUAECs (Fig. 4A). This was accompanied by decreased cell viability, proliferation and survival (Fig. 4B–D). In contrast, hyperoxia-exposed HUAECs treated with varying doses of TC14012 had improved cell viability, increased cell proliferation, and survival (Fig. 4B–D). This suggests that CXCR7 may play an important role in preventing hyperoxia-induced systemic vascular endothelial cell injury.

**Figure 5:**
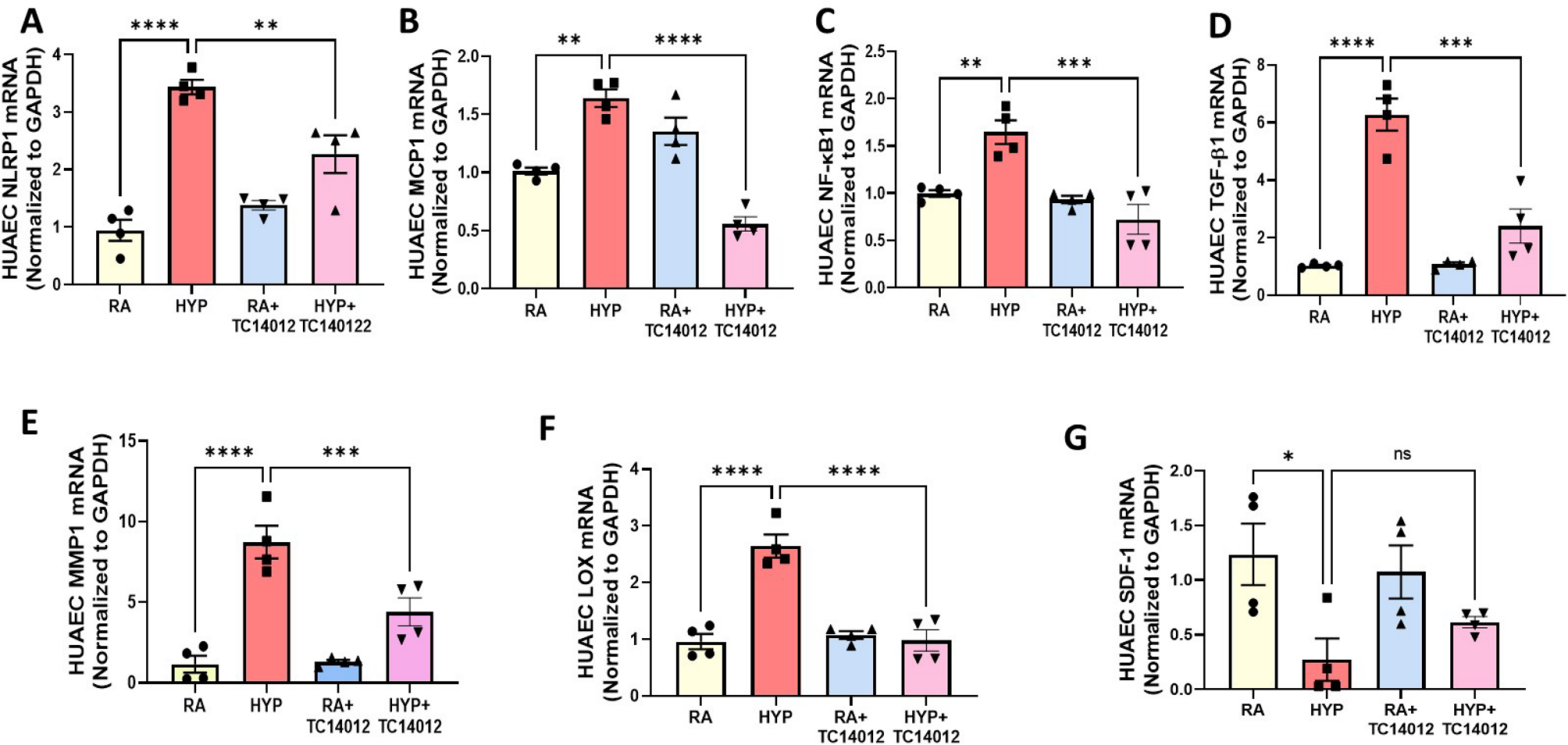
CXCR7 treatment ameliorates pro-inflammatory markers in hyperoxia-exposed HUAECs. Normoxia and hyperoxia-exposed HUAECs were treated with TC14012, which reduced the gene expression of (**A**) NLRP1 (**B**) MCP-1 (**C**) NF-кB1 (**D**) TGF-β1 (**E**) MMP1, (**F**) LOX (**G**) SDF-1 in hyperoxia-exposed endothelial cells. n = 4/group, data are mean ± SEM, Two-way ANOVA with Tukey’s multiple comparisons test. *p < 0.05, **p < 0.01, ***p < 0.001, ****p < 0.0001; RA = normoxia versus HYP = hyperoxia versus RA + TC14012 = normoxia treated with TC14012 versus HYP + TC14012 = hyperoxia-exposed treated with TC14012; all experiments were performed in quadriplicate.

Given that neonatal hyperoxia is associated with aberrant cardiovascular inflammation and fibrosis, we next evaluated the expression of key inflammatory, pro-fibrotic and matrix remodeling genes in HUAECs. As expected, hyperoxia increased the expression of several genes related with inflammation such as NLR pyrin domain containing (NLRP1), 3.4-fold; monocyte chemoattractant protein-1 (MCP-1), 1.6-fold; nuclear factor kappa B1 subunit (NF-кB1), 1.6-fold; TGF-β1, 6.2-fold; (Fig. 5A–D), matrix remodeling genes such as matrix metalloproteinase-1 (MMP-1), eightfold; lysyl oxidase (LOX), 2.7-fold; (Fig. 5E–F) and decreased gene expression of SDF-1, fourfold (Fig. 5G). Treatment with TC14012 partially reversed this effect, suggesting that CXCR7 agonism may blunt the inflammatory response of the hyperoxia-exposed systemic vascular endothelial cells.

### CXCR7 agonism decreases hyperoxia-induced inflammation in human coronary endothelial cells (HCAECs)

**Figure 6:**
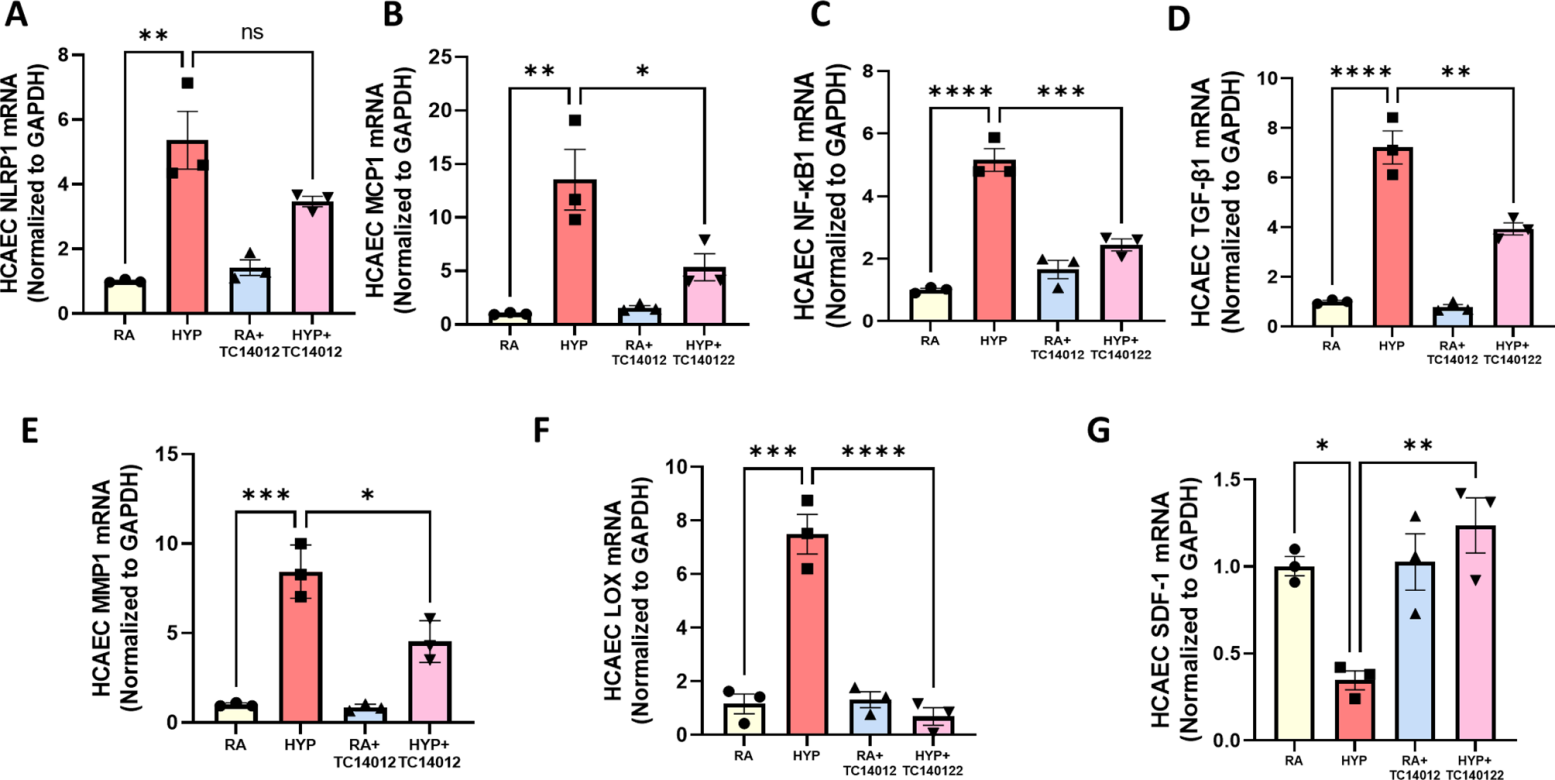
CXCR7 decreases proinflammatory response in hyperoxia-exposed HCAECs. Treatment with TC14012 of hyperoxia-exposed HCAECs reduced the gene expression of (**A**) NLRP1 (**B**) MCP-1 (**C**) NF-кB1 (**D**) TGF-β1 (**E**) MMP1 (**F**) LOX (**G**) SDF-1. n = 3/group, data are mean ± SEM, Two-way ANOVA with Tukey’s multiple comparisons test. *p < 0.05, **p < 0.01, ***p < 0.001, ****p < 0.0001; RA = normoxia versus HYP = hyperoxia versus RA + TC14012 = normoxia treated with TC14012 versus HYP +TC14012 = hyperoxia-exposed treated with TC14012; all experiments were performed in triplicate.

Given the beneficial effects of CXCR7 agonism in our in vivo model of cardiac dysfunction, we also investigated the effect of CXCR7 agonism in hyperoxia-exposed HCAECs. Consistent with our findings in the HUAECs, hyperoxia exposure of the HCAECs increased the expression of several genes related with inflammation, NLRP1 (5.3-fold), MCP-1 (13-fold), NF-кB1 (5.2-fold), TGF-β1 (6.2-fold) (Fig. 6A–D), matrix remodeling MMP1 (ninefold), LOX (7.2-fold) (Fig. 6E,F), and decreased gene expression of SDF-1 (threefold) (Fig. 6G). This was partially prevented by CXCR7 agonism in the coronary endothelial cells, suggesting that the CXCR7 attenuates hyperoxia-induced upregulation of pro-inflammatory, pro-fibrotic and matrix remodeling pathways.

## Discussion

Evidence from experimental and early clinical studies show that preterm born survivors have persistently impaired cardiovascular function^9,11,33,34^. However, the pathogenic mechanisms that result in this cardiovascular dysfunction in preterm born survivors are poorly understood. In adult cardiovascular diseases, CXCR7 has been shown to exert an atheroprotective, cardioprotective and antithrombotic role through a wide range of cells such as endothelial cells, inflammatory cells, platelets, fibroblasts and cardiomyocytes^35^. In this study, we demonstrate a novel role for CXCR7 in modulating systemic vascular stiffness and cardiac function in juvenile rats exposed to neonatal hyperoxia. In vivo, we show that neonatal hyperoxia exposure induces systemic vascular stiffness and LV dysfunction. This was accompanied by decreased CXCR7 and increased TGF-β1 levels in the aorta and LV of these hyperoxia-exposed rats. Early administration of a CXCR7 agonist partially prevented neonatal hyperoxia-induced systemic vascular stiffness and preserved LV function. This improvement was associated with decreased aortic and LV TGF-β1 levels. In vitro, we show that hyperoxia exposure decreases CXCR7 expression in systemic vascular endothelial cells. CXCR7 agonism improved cell survival and proliferation in hyperoxia-exposed systemic vascular endothelial cells. Additionally, CXCR7 agonism decreased TGF-β1 levels in hyperoxia-exposed systemic vascular and coronary artery endothelial cells. Taken together, these findings suggest that pharmacological CXCR7 agonism may prevent the cardiovascular consequences of preterm birth by modulating TGF-β1 expression.

Hyperoxia exposure in preterm infants is a well-known cause of multiorgan damage in preterm infants^36–38^. Additionally, the link between hyperoxia and vascular stiffness is consistently shown even in adults exposed to hyperoxia, where healthy human subjects exposed to hyperoxia with 100% normobaric oxygen for 30 min have increased arterial stiffness, as manifested by increased pulse wave augmentation index and elevated biochemical markers of oxidative stress^39^. In our present study, we used the neonatal hyperoxia rodent model, which recapitulates the condition of prematurity where antioxidant defense is deficient and supplemental oxygen renders the vasculature vulnerable to oxidative damage. Hyperoxia exposure increases oxidative stress and leads to endothelial dysfunction, a well-recognized marker of cardiovascular disease^40,41^. Intracellular signals from injured endothelial cells interact with underlying vascular smooth muscle cells triggering extracellular matrix remodeling and fibrosis^42^.

In our present rodent study, we show a significant reduction in aortic and LV CXCR7 and SDF-1 levels in juvenile rats exposed to neonatal hyperoxia. These findings are consistent with our prior studies showing decreased SDF-1 levels in the lungs of neonatal hyperoxia-exposed rats^43^. Interestingly, in injured arteries of humans and mice, endothelial CXCR7 was upregulated^26^. Single-cardiomyocyte RNA sequencing of human heart specimens also demonstrated increased CXCR7 expression in heart failure patients^44^. Additionally, SDF-1 mRNA is selectively induced in adult rodents with myocardial infarction^45^. It is plausible that the reduction in SDF-1 and CXCR7 levels seen in our neonatal hyperoxia model contributes to suboptimal cardiovascular repair during a vulnerable phase of development.

To further explore the potential protective role of CXCR7 in systemic vascular stiffness, we administered a CXCR7 agonist, TC14012 to neonatal rats exposed to hyperoxia. Whereas, placebo treated animals, had a significant increase in vascular stiffness with hyperoxia exposure, there was no difference in PWV between the room air and hyperoxic groups who received treatment with TC14012. The exact mechanisms by which CXCR7 activation decreases vascular stiffness are unclear. Prior studies indicate that CXCR7 agonism inhibits NLRP3 inflammasome signaling^46^, activates p38 mitogen-activated protein kinase pro-survival signaling pathways^47^, and promotes endothelial progenitor cell function^48^. Endothelial dysfunction is a known precursor to vascular remodeling^26,30^. CXCR7 agonism reduces atherosclerotic lesions in apolipoprotein E (ApoE^−^/^**−**^) mice fed a high fat diet and promotes endothelial repair in a diabetic limb ischemia model^46,48^. High oxygen supplementation after birth increases oxidative stress and leads to endothelial injury and dysfunction^40,41^. Our in vitro evidence in hyperoxia-exposed systemic vascular endothelial cells demonstrating that CXCR7 agonism increases endothelial cell proliferation and survival, and decreases the expression of endothelial proinflammatory, profibrotic and matrix remodeling markers suggests that CXCR7 endothelial protection prevents hyperoxia-exposure induced vascular stiffness. Our study findings are consistent with other studies showing that CXCR7 knockdown inhibits human umbilical vein endothelial cell survival and proliferation. Moreover, CXCR7 overexpression improves cell migration and angiogenesis^49^.

Another important finding of our study is that CXCR7 activation distinctly preserved long-term LV function in neonatal hyperoxia-exposed rats. CXCR7 is abundantly expressed in cardiomyocytes and cardiomyocyte specific-CXCR7 null mice showed more cardiac dysfunction than controls following myocardial infarction^44^. Our present findings are supportive of prior studies showing that increased CXCR7 surface expression on circulating platelets correlates with recovery of cardiac function in patients with acute coronary syndrome and ST-segment elevation myocardial infarction^50,51^. Moreover, CXCR7 agonism provides cardiac protection after myocardial infarction by decreasing infarct size, improving cardiac function and promoting angiogenesis^52^.

Vascular inflammation, fibrosis and extracellular matrix remodeling are important contributors to vascular remodeling and cardiac dysfunction^53–56^. We and others have shown that neonatal hyperoxia induces cardiovascular fibrosis and remodeling, however the mechanisms have been less explored^14,53,57^. Consistent with our previous finding, we demonstrate that hyperoxia caused cardiovascular fibrosis in juvenile rats. In contrast, treatment with a CXCR7 agonist preserved cardiovascular function and decreased TGF-β levels in the myocardium and aorta of hyperoxia exposed animals. TGF-β is a major driver of fibrosis. Endothelial CXCR7 overexpression attenuates TGF-β-induced EMT in rodent lung fibrosis models^58^. CXCR7 agonism also reduces fibrosis by suppressing Jagged/Notch and Wnt/β-catenin pro-fibrotic signaling pathways^27,28,49^. We speculate that our current findings of improved cardiovascular function are in part secondary to decreased TGF-β1 profibrotic signaling.

Our study has strengths and limitations. To the best of our knowledge, this is the first study to unveil the role of CXCR7 in neonatal hyperoxia-induced vascular dysfunction in juvenile rats, similar to that seen in preterm survivors thus suggesting the clinically relevant nature of the study. Using both in vivo and in vitro methods, we show that CXCR7 agonism is a potential therapeutic strategy to treat the vascular consequences of preterm birth where fibrosis is a key culprit. Our study has specific limitations as well. We used a high concentration of oxygen to induce the vascular phenotype in our experimental model, which is not routinely used clinically in preterm infants. However, this concentration of oxygen induces vascular dysfunction as seen in preterm born adults. Although we show that hyperoxia plays a role in vascular dysfunction, we recognize that vascular dysfunction is multifactorial in etiology, and hence this study would need to be evaluated in other experimental models. Also, while we show that CXCR7 expression is significantly decreased in remodeled aortas and systemic endothelial cells, further mechanistic studies exploring the post transcriptional effects of CXCR7 agonism on inflammation, fibrosis and matrix remodeling would be important. Prior work has shown that CXCR7 and CXCR4 can form CXCR7/CXCR4 homo-heterodimers and the cross-talk between them can modulate SDF-1^59^. Research into the interaction between CXCR7 and CXCR4 is warranted in the next study. Additionally, TC14012 dosing, its efficacy and off therapeutic targets in preterm neonates would need to be further studied. It is also important to note that while our current study showed improvement in the profibrotic, proinflammatory markers in the HCAECs, these cells were obtained from the adult coronary arteries, which may not reflect the behavior of the neonatal vasculature. Previous studies have also shown that CXCR7 overexpression promotes homing of mesenchymal stem cells and endothelial progenitor cells to the injured myocardium^59,60^. Although beyond the scope of this study, CXCR7 may have potentially improved cardiovascular function by stem cell-mediated mechanisms. The role of SDF-1 in injury and repair has also yielded conflicting results. While SDF-1 has been shown to promote angiogenesis, and improve cardiac function, some studies suggest it may potentiate fibrosis^61–63^. In our study, while hyperoxia exposure decreased SDF-1 expression in HUAECs and HCAECs, there was a differential restoration of SDF-1 to normoxic levels with CXCR7 agonism. This discrepancy may be secondary to differential effect of TC14012 on various vascular beds, the relative surface exposure of CXCR7 in these vascular beds, and the difference in donor age of the endothelial cells.

In summary, in this study we report a novel role for CXCR7 in neonatal hyperoxia-induced vascular stiffness and cardiac dysfunction in a rat model of adults born preterm. We speculate that strategies which modulate CXCR7 signaling may improve systemic vascular stiffness and cardiac dysfunction in preterm born survivors. Further studies are warranted to assess the specific mechanisms by which CXCR7 activation preserves endothelial function and vascular function in preterm born survivors. Targeting CXCR7 signaling may be a potential strategy to abrogate the vascular consequence of preterm birth.

## Methods

### Ethics statement

The Animal Care and Use Committee at the University of Miami Miller School of Medicine approved the protocol. We performed this study in strict accordance with the recommendations in the Guide for the Care and Use of Laboratory Animals of the National Institutes of Health and ARRIVE guidelines. All surgery was performed under isoflurane anesthesia, and every effort was made to minimize suffering.

### Experimental model

#### *In vivo* experiment 1: Neonatal Hyperoxia model and its effect on CXCR7

Pregnant Sprague–Dawley rats were obtained from Charles River Laboratories (Wilmington, MA) and housed with food and water available ad libitum at constant temperature (25 °C) under 12:12 light/dark cycle. The rat pups (N = 22) were housed in a plexiglass chamber with continuous O_2_ exposure and monitoring from postnatal day 1 to 21, that was briefly interrupted for animal care (< 10 min/day). Oxygen level inside the chamber was monitored daily with a Maxtec Oxygen Analyzer (Model OM-25; Maxtec, Salt Lake City, Utah). Mothers were rotated every 48 h between room air and hyperoxia chambers to prevent damage to their lungs. Litter size was adjusted to 10–12 pups to control for the effect of litter size on growth and nutrition. The rats were recovered in room air for additional 3 weeks. Both male and female rats were studied at 6 weeks.

#### *In vivo* experiment 2: Assessment of CXCR7 agonist, TC14012 on systemic vascular remodeling and cardiovascular dysfunction

Sprague–Dawley rat pups (N = 60) were assigned to room air (RA; 21% Oxygen) or hyperoxia (HYP; 85% Oxygen) were randomly assigned to receive intraperitoneal (IP) injection of CXCR7 agonist, TC14012, 5 mg/kg (Cayman Chemical, Ann Arbor Michigan), or phosphate buffered saline as placebo (PL) every third day from postnatal day 1 to 21. Litter size was adjusted to 10–12 pups to control for the effect of litter size on growth and nutrition. The rats were recovered in room air for additional 3 weeks. Both male and female rats were studied at 6 weeks.

### *In vivo* systemic vascular dysfunction-pulse wave doppler and cardiac echocardiography

Pulse wave velocity (PWV) was measured by high-resolution Doppler ultrasonography (Vevo2100 imaging system, VisualSonics, Toronto, Canada) in 6 week rat abdominal aorta as previously described^14,64^. The determination of PWV is based on the transit time method utilized to determine the difference in arrival times of a Doppler pulse wave at two locations along the aorta a known distance apart. The distance between the two locations along the aorta is divided by the difference in arrival times and is expressed in m/s. All measurements were obtained in triplicate. In addition, LV fractional shortening and ejection fraction were assessed using M-mode and cardiac output, stroke volume and end systolic volume were determined using B-mode. The average of 3–5 representative cardiac cycles was used to calculate the data using VevoLab 1.7 software. Ultrasound procedures were performed on 2–3% isoflurane-anesthetized mice, controlled core body temperatures (37 ± 1 °C).

### Localization of CXCR7 in the aorta

CXCR7 localization in aorta sections was evaluated by immunohistochemistry and double immunofluorescence staining. Briefly, aortic sections were incubated with CXCR7 polyclonal antibody (1:100; Ab 38089, Abcam, Cambridge, MA) overnight at 4 °C. The following day, sections were incubated with biotinylated horseradish peroxidase (HRP)-conjugate goat anti-rabbit secondary antibody (A5420, 1:200; Sigma-Aldrich, St. Louis, MO) for 1 h. The sections were then incubated for 45 min with the streptavidin-HRP (Vector Laboratories Inc., Burlingame, CA). Peroxidase activity was detected with 3, 3′-Diaminobenzidine (Vector Laboratories Inc.). Sections were counterstained with hematoxylin, dehydrated, and mounted with Richard-Allan cytoseal XYL (# 8312-4, ThermoFisher Scientific Waltham, MA). Quantification of immunohistochemistry for CXCR7 positive staining was analyzed by calculating the integration optical density value of positive staining using Image J. To determine whether CXCR7 is expressed on endothelial cells, double immunofluorescence staining of rat abdominal aortas with CXCR7 (1:500; Ab 38089, Abcam, Cambridge, MA) and Von Willebrand Factor (VWF; 1:100; Dako, Carpinteria, CA) antibody was performed. Images were captured with a fluorescent microscope (Leica DMI 6000, Mannheim, Germany) at 20× magnification.

### Western blot

Aortic, cardiac and HUAEC protein expression of CXCR7 (1:500; Ab 138509, Abcam) and SDF-1 (1:500; #3740, Cell Signaling Technology, Danvers, MA) was performed by Western blot as previously described^14,65^. Band intensity was analyzed by a Quantity One Imaging Analysis Program (Bio-Rad, Hercules, CA) and normalized by β-actin (1:10,000; A5441, Sigma Aldrich, St. Louis, MO).

### Real-time PCR

The gene expression of CXCR7, TGF-β1, Col1a2 and GAPDH was determined by real-time RT-PCR as previously described^53^. RNA from aortic tissue was extracted (miRNeasy Mini Kit; Cat#217004; Qiagen Inc,Valencia, CA) and reverse transcribed (Superscript VIVILO Master Mix; Cat# 11766,050, ThermoFisher, Cambridge, MA). Real time RT-PCR using gene specific primers, and TaqMan Fast Advanced Master Mix (Cat #4444554, Applied Biosystems, Foster City, CA) was performed on an ABI Fast 7500 system (Applied Biosystems). Primers for CXCR7 (Rn00584358_m1), TGF-β1 (Rn00572010_m1), Col1a2 (Rn00584426_m1), GAPDH (Cat#Rn99999916_s1) were used. Table S1 lists the human primers for NLRP1, MCP-1, NF-кB1, MMP1, LOX, SDF-1, TGF-β1 and GAPDH used. The relative mRNA expression of the specific genes was normalized to GAPDH.

### Cell culture and treatment

HUAECs (202-05n, Cell Applications, San Diego, CA) and HCAECs (300-05a, Cell Applications) were used for in vitro experiments. The cells were cultured according to manufacturer instructions. Briefly, HUAECs were cultured in human MesoEndo growth medium (# 210470 + 212-GS, Cell Applications, San Diego, CA) to passage 3–6. Several groups of HUAECs were seeded into 100-mm dishes at a density of 1 × 10^6^ cells/dish and incubated overnight. Cells were then cultured in normoxia (21% O_2_, 5% CO_2_) or hyperoxia (95% O_2_, 5% CO_2_) conditions for 24, 48 and 72 h. CXCR7 protein expression was assessed by Western Blot analysis. In another subset cultured cells were serum deprived for 24 h, incubated with placebo (0.25% DMSO/PBS) or TC-14012 (50 ng/ml), based on our pilot studies and exposed to hyperoxia (95% O_2_, 5% CO_2_) for 24 h and recovered in normoxia for an additional 24 h. HCAECs were cultured to passages 3–5 and were serum deprived for 24 h, incubated with placebo (0.25% DMSO/PBS) or TC-14012 (50 ng/ml) and exposed to hyperoxia (95% O_2_, 5% CO_2_) for 24 h and recovered in normoxia for an additional 24 h.

### MTT assay

The effect of CXCR7 agonism on HUAECs viability was determined by MTT assay (Sigma-Aldrich, St. Louis, MO). HUAECs (1 × 10^4^ cells/well) cultured overnight in 96-well plates and treated with the CXCR7 agonist, TC-14012 at 10–100 ng/ml (Cayman Chemicals, Ann Arbor, Michigan) were exposed to normoxia (21% O_2_, 5% CO_2_) or hyperoxia (95% O_2_, 5% CO_2_) for 12 h. MTT labeling solution was added 4 h before the end of the incubation. The plate was then incubated at 37 °C for 5 min following, which was transferred to a plate reader (SpectraMax Plus 384 Microplate Reader; San Jose, CA) and absorbance was measured at a wavelength of 550 nm. The experiment was performed in triplicate.

### HUAEC cell proliferation and cell death

For proliferation and cell death studies, cells were seeded in LabTek slides at 40,000 cells/well and incubated overnight in normoxic conditions. Cells were then treated with placebo (0.25% DMSO/PBS) or TC-14012 (50 ng/ml) and exposed to hyperoxia (95% O_2_, 5% CO_2_) for an additional 18 h. The cells were then fixed with 4% paraformaldehyde and assays for proliferation (Ki67 immunofluorescence staining) and cell death (TUNEL assay; Thermo Fisher Scientific, Waltham, MA) performed as previously described^66^. Five randomly selected fields were photographed, and the number of proliferative or apoptotic nuclei, as well as the total number of nuclei was counted per HPF. The proliferative or apoptotic index was obtained by means of the formula: number of proliferative or apoptotic cells per field / total number of cells per field.

### Multiplex protein quantification

Flash frozen LV tissues were homogenized in RIPA buffer (Cat# sc-24948, Santa Cruz Biotechnology, Santa Cruz, CA) and centrifuged at 15,000 rpm for 15 min at 4 °C. The protein concentration of the supernatant was measured by BCA protein assay (Cat# PI2322 ThermoFisher Scientific, Waltham, MA). All the samples were then diluted for target protein concentration of 100 mg/ml. These protein samples were outsourced to Eve Technologies (Calgary, AB, Canada) for TGF-β1-3 protein concentration using the Rat TGF-β 3-Plex Discovery Assay, a multiplex immunoassay. TGF-β concentration was expressed as picograms/milliliter (pg/ml).

### Statistics

For all experiments, n refers to the number of individual rats or individual culture plates. All data are expressed as mean ± SEM. *P* values were calculated using Student's *t*-tests (two group comparison) and Two-way ANOVA with Tukey’s multiple comparisons test (four group comparison). All analyses were performed using commercially available statistical software packages (GraphPad Prism version 8.3 for Windows, GraphPad Software, San Diego, CA).

### Supplementary Information

Supplementary Information.

## Data Availability

The data that support the findings of this study are available from the corresponding author upon reasonable request.

## References

[1] Lewandowski AJ, Augustine D, Lamata P (2013). Preterm heart in adult life: Cardiovascular magnetic resonance reveals distinct differences in left ventricular mass, geometry, and function. Circulation.

[2] Chehade H, Simeoni U, Guignard JP, Boubred F (2018). Preterm birth: Long term cardiovascular and renal consequences. Curr. Pediatr. Rev..

[3] Norman M (2013). Premature birth: Implications for cardiovascular health. Future Cardiol..

[4] Crump C (2015). Birth history is forever: Implications for family medicine. J. Am. Board Fam. Med. JABFM.

[5] Raju TNK, Pemberton VL, Saigal S, Blaisdell CJ, Moxey-Mims M, Buist S (2017). Long-term healthcare outcomes of preterm birth: An executive summary of a conference sponsored by the national institutes of health. J. Pediatr..

[6] Hurst JR, Beckmann J, Ni Y (2020). Respiratory and cardiovascular outcomes in survivors of extremely preterm birth at 19 years. Am. J. Respir. Crit. Care Med..

[7] Burchert H, Lewandowski AJ (2019). Preterm birth is a novel, independent risk factor for altered cardiac remodeling and early heart failure: Is it time for a new cardiomyopathy?. Curr. Treat. Opt. Cardiovasc. Med..

[8] de Jong F, Monuteaux MC, van Elburg RM, Gillman MW, Belfort MB (2012). Systematic review and meta-analysis of preterm birth and later systolic blood pressure. Hypertension.

[9] Crump C, Howell EA, Stroustrup A, McLaughlin MA, Sundquist J, Sundquist K (2019). Association of preterm birth with risk of ischemic heart disease in adulthoodassociation of preterm birth with risk of ischemic heart disease in adulthood association of preterm birth with risk of ischemic heart disease in adulthood. JAMA Pediatr..

[10] Hovi P, Turanlahti M, Strang-Karlsson S (2011). Intima-media thickness and flow-mediated dilatation in the Helsinki study of very low birth weight adults. Pediatrics.

[11] Carr H, Cnattingius S, Granath F, Ludvigsson JF, Edstedt Bonamy AK (2017). Preterm birth and risk of heart failure up to early adulthood. J. Am. Coll. Cardiol..

[12] Buczynski BW, Maduekwe ET, O'Reilly MA (2013). The role of hyperoxia in the pathogenesis of experimental BPD. Semin. Perinatol..

[13] Richter AE, Bos AF, Huiskamp EA, Kooi EMW (2019). Postnatal cerebral hyperoxia is associated with an increased risk of severe retinopathy of prematurity. Neonatology.

[14] Benny M, Hernandez DR, Sharma M (2020). Neonatal hyperoxia exposure induces aortic biomechanical alterations and cardiac dysfunction in juvenile rats. Physiol. Rep..

[15] Mivelaz Y, Yzydorczyk C, Barbier A (2011). Neonatal oxygen exposure leads to increased aortic wall stiffness in adult rats: A doppler ultrasound study. J. Dev. Orig. Health Dis..

[16] Ravizzoni Dartora D, Flahault A, Pontes CNR (2022). Cardiac left ventricle mitochondrial dysfunction after neonatal exposure to hyperoxia: Relevance for cardiomyopathy after preterm birth. Hypertension.

[17] Noels H, Weber C, Koenen RR (2019). Chemokines as therapeutic targets in cardiovascular disease. Arterioscler. Thromb. Vasc. Biol..

[18] Dusi V, Ghidoni A, Ravera A, De Ferrari GM, Calvillo L (2016). Chemokines and heart disease: A network connecting cardiovascular biology to immune and autonomic nervous systems. Mediat. Inflamm..

[19] Deloukas P, Kanoni S, Willenborg C (2013). Large-scale association analysis identifies new risk loci for coronary artery disease. Nat. Genet..

[20] Chen D, Xia Y, Zuo K (2015). Crosstalk between SDF-1/CXCR4 and SDF-1/CXCR7 in cardiac stem cell migration. Sci. Rep..

[21] Balabanian K, Lagane B, Infantino S (2005). The chemokine SDF-1/CXCL12 binds to and signals through the orphan receptor RDC1 in T lymphocytes. J. Biol. Chem..

[22] Zhang M, Qiu L, Zhang Y, Xu D, Zheng JC, Jiang L (2017). CXCL12 enhances angiogenesis through CXCR7 activation in human umbilical vein endothelial cells. Sci. Rep..

[23] Gerrits H, van Ingen Schenau DS, Bakker NE (2008). Early postnatal lethality and cardiovascular defects in CXCR7-deficient mice. Genesis.

[24] Wei S-T, Huang YC, Hsieh M-L (2020). Atypical chemokine receptor ACKR3/CXCR7 controls postnatal vasculogenesis and arterial specification by mesenchymal stem cells via Notch signaling. Cell Death Disease.

[25] Sierro F, Biben C, Martínez-Muñoz L (2007). Disrupted cardiac development but normal hematopoiesis in mice deficient in the second CXCL12/SDF-1 receptor, CXCR7. Proc. National Acad. Sci. U. S. A..

[26] Hao H, Hu S, Chen H (2017). Loss of endothelial CXCR7 impairs vascular homeostasis and cardiac remodeling after myocardial infarction: Implications for cardiovascular drug discovery. Circulation.

[27] Ding BS, Cao Z, Lis R (2014). Divergent angiocrine signals from vascular niche balance liver regeneration and fibrosis. Nature.

[28] Cao Z, Lis R, Ginsberg M (2016). Targeting of the pulmonary capillary vascular niche promotes lung alveolar repair and ameliorates fibrosis. Nat. Med..

[29] Yu S, Crawford D, Tsuchihashi T, Behrens TW, Srivastava D (2011). The chemokine receptor CXCR7 functions to regulate cardiac valve remodeling. Dev. Dyn. Off. Publ. Am. Assoc. Anat..

[30] Ding H, Triggle CR (2005). Endothelial cell dysfunction and the vascular complications associated with type 2 diabetes: Assessing the health of the endothelium. Vasc. Health Risk Manag..

[31] Blacher J, Safar ME (2005). Large-artery stiffness, hypertension and cardiovascular risk in older patients. Nat. Clin. Pract. Cardiovasc. Med..

[32] Kim HL, Kim SH (2019). Pulse wave velocity in atherosclerosis. Front. Cardiovasc. Med..

[33] Bates ML, Levy PT, Nuyt AM, Goss KN, Lewandowski AJ, McNamara PJ (2020). Adult cardiovascular health risk and cardiovascular phenotypes of prematurity. J. Pediatr..

[34] Crump C, Winkleby MA, Sundquist K, Sundquist J (2011). Risk of hypertension among young adults who were born preterm: A Swedish national study of 636,000 births. Am. J. Epidemiol..

[35] Duval V, Alayrac P, Silvestre JS, Levoye A (2022). Emerging roles of the atypical chemokine receptor 3 (ACKR3) in cardiovascular diseases. Front. Endocrinol. (Lausanne).

[36] Bhandari V (2010). Hyperoxia-derived lung damage in preterm infants. Semin. Fetal Neonatal Med..

[37] Reich B, Hoeber D, Bendix I, Felderhoff-Mueser U (2016). Hyperoxia and the immature brain. Dev. Neurosci..

[38] Hu Y, Xie L, Yu J, Fu H, Zhou D, Liu H (2019). Inhibition of microRNA-29a alleviates hyperoxia-induced bronchopulmonary dysplasia in neonatal mice via upregulation of GAB1. Mol. Med..

[39] Vukovic J, Modun D, Budimir D (2009). Acute, food-induced moderate elevation of plasma uric acid protects against hyperoxia-induced oxidative stress and increase in arterial stiffness in healthy humans. Atherosclerosis.

[40] Pennathur S, Heinecke JW (2007). Oxidative stress and endothelial dysfunction in vascular disease. Curr. Diabetes Rep..

[41] Fujinaga H, Baker CD, Ryan SL (2009). Hyperoxia disrupts vascular endothelial growth factor-nitric oxide signaling and decreases growth of endothelial colony-forming cells from preterm infants. Am. J. Physiol. Lung Cell. Mol. Physiol..

[42] Li M, Qian M, Kyler K, Xu J (2018). Endothelial-vascular smooth muscle cells interactions in atherosclerosis. Front. Cardiovasc. Med..

[43] Guerra K, Bryan C, Dapaah-Siakwan F (2019). Intra-tracheal administration of a naked plasmid expressing stromal derived factor-1 improves lung structure in rodents with experimental bronchopulmonary dysplasia. Respir. Res..

[44] Ishizuka M, Harada M, Nomura S (2021). CXCR7 ameliorates myocardial infarction as a beta-arrestin-biased receptor. Sci. Rep..

[45] Pillarisetti K, Gupta SK (2001). Cloning and relative expression analysis of rat stromal cell derived factor-1 (SDF-1): SDF-1 α mRNA is selectively induced in rat model of myocardial infarction. Inflammation.

[46] Qiu L, Zhang M, Zhang S (2020). Activation of CXCR7 promotes endothelial repair and reduces the carotid atherosclerotic lesions through inhibition of pyroptosis signaling pathways. Aging Cell.

[47] Zhang X-Y, Su C, Cao Z (2014). CXCR7 upregulation is required for early endothelial progenitor cell-mediated endothelial repair in patients with hypertension. Hypertension.

[48] Wang K, Sun S, Zhang G (2022). CXCR7 agonist TC14012 improves angiogenic function of endothelial progenitor cells via activating Akt/eNOS pathway and promotes ischemic angiogenesis in diabetic limb ischemia. Cardiovasc. Drugs Ther..

[49] Shen M, Feng Y, Wang J, Yuan Y, Yuan F (2020). CXCR7 inhibits fibrosis via Wnt/beta-catenin pathways during the process of angiogenesis in human umbilical vein endothelial cells. Biomed. Res. Int..

[50] Rath D, Chatterjee M, Borst O (2014). Expression of stromal cell-derived factor-1 receptors CXCR4 and CXCR7 on circulating platelets of patients with acute coronary syndrome and association with left ventricular functional recovery. Eur. Heart J..

[51] Rath D, Chatterjee M, Meyer L (2018). Relative survival potential of platelets is associated with platelet CXCR4/CXCR7 surface exposure and functional recovery following STEMI. Atherosclerosis.

[52] Zhang S, Yue J, Ge Z, Xie Y, Zhang M, Jiang L (2020). Activation of CXCR7 alleviates cardiac insufficiency after myocardial infarction by promoting angiogenesis and reducing apoptosis. Biomed. Pharmacother..

[53] Batlahally S, Franklin A, Damianos A (2020). Soluble Klotho, a biomarker and therapeutic strategy to reduce bronchopulmonary dysplasia and pulmonary hypertension in preterm infants. Sci. Rep..

[54] Mian M, Bigras JL, Fernandes R (2017). [LB.03.01] Long term cardiac structure correlates with adverse perinatal complications in young adults born preterm. J. Hypertens..

[55] Mian MOR, He Y, Bertagnolli M (2019). TLR (Toll-Like Receptor) 4 antagonism prevents left ventricular hypertrophy and dysfunction caused by neonatal hyperoxia exposure in rats. Hypertension.

[56] Harvey A, Montezano AC, Lopes RA, Rios F, Touyz RM (2016). Vascular fibrosis in aging and hypertension: Molecular mechanisms and clinical implications. Can. J. Cardiol..

[57] Bertagnolli M, Huyard F, Cloutier A (2014). Transient neonatal high oxygen exposure leads to early adult cardiac dysfunction, remodeling, and activation of the renin-angiotensin system. Hypertension.

[58] Guan S, Zhou J (2017). CXCR7 attenuates the TGF-beta-induced endothelial-to-mesenchymal transition and pulmonary fibrosis. Mol. BioSyst..

[59] Chen D, Xia Y, Zuo K (2015). Crosstalk between SDF-1/CXCR4 and SDF-1/CXCR7 in cardiac stem cell migration. Sci. Rep..

[60] Shao Y, Zhou F, He D, Zhang L, Shen J (2019). Overexpression of CXCR7 promotes mesenchymal stem cells to repair phosgene-induced acute lung injury in rats. Biomed. Pharmacother..

[61] Yamaguchi J, Kusano KF, Masuo O (2003). Stromal cell-derived factor-1 effects on ex vivo expanded endothelial progenitor cell recruitment for ischemic neovascularization. Circulation.

[62] Askari AT, Unzek S, Popovic ZB (2003). Effect of stromal-cell-derived factor 1 on stem-cell homing and tissue regeneration in ischaemic cardiomyopathy. Lancet.

[63] Xu J, Mora A, Shim H, Stecenko A, Brigham KL, Rojas M (2007). Role of the SDF-1/CXCR4 axis in the pathogenesis of lung injury and fibrosis. Am. J. Respir. Cell Mol. Biol..

[64] Lee L, Cui JZ, Cua M (2016). Aortic and cardiac structure and function using high-resolution echocardiography and optical coherence tomography in a mouse model of Marfan syndrome. PLoS One.

[65] Hummler SC, Rong M, Chen S, Hehre D, Alapati D, Wu S (2013). Targeting glycogen synthase kinase-3beta to prevent hyperoxia-induced lung injury in neonatal rats. Am. J. Respir. Cell Mol. Biol..

[66] Dapaah-Siakwan F, Zambrano R, Luo S (2019). Caspase-1 inhibition attenuates hyperoxia-induced lung and brain injury in neonatal mice. Am. J. Respir. Cell Mol. Biol..

