# Supplementary material for: Protective role of CXCR7 activation in neonatal hyperoxia-induced systemic vascular remodeling and cardiovascular dysfunction in juvenile rats
Source: Sci Rep. 2023 Nov 9;13:19538. doi: 10.1038/s41598-023-46422-3 (PMC10636097; doi:10.1038/s41598-023-46422-3)
Supplement: Supplementary file 1 — Supplementary Information. [file 41598_2023_46422_MOESM1_ESM.pdf]

SUPPLEMENTAL MATERIAL

Protective role of CXCR7 Activation in Neonatal Hyperoxia-Induced Systemic Vascular Remodeling and Cardiovascular Dysfunction in Juvenile Rats

Merline Benny[1,2], Mayank Sharma[1,2], Shathiyah Kulandavelu[1,3], PingPing Chen[1,2], Runxia Tian[1,2], Sydne Ballengee[1,2], Jiang Huang[1,2], Amanda F. Levine[1,2], Matteo Claure[1,2], Augusto F. Schmidt[1,2], Roberto I. Vazquez-Padron[4], Claudia O. Rodrigues[3,5], Shu Wu[1,2], Omaida C. Velazquez[4], Karen C. Young[1,2,3]

[1]Department of Pediatrics, [2]Batchelor Children's Research Institute, [3]The Interdisciplinary Stem Cell Institute, [4]Department of Surgery, [5]Department of Molecular and Cellular Pharmacology, University of Miami Miller School of Medicine, Florida

*Correspondence
E-mail: mxk968@med.miami.edu

Table S1. Primer sequences for PCR amplification

	Sequence (5' -> 3')	Length	Amplicon Size(bp)
Human NLRP1	F: GCAGTGCTAATGCCCTGGAT	20	136
	R: GAGCTTGGTAGAGGAGTGAGG	21	
Human MCP-1	F: GCAAGTGTCCCAAAGAAGCT	20	150
	R: TGGGTTGTGGAGTGAGTGTT	20	
Human NF-κB1	F: AACAGAGAGGATTTCGTTTCCG	21	104
	R: TTTGACCTGAGGGTAAGACTTCT	23	
Human MMP-1	F: AAAATTACACGCCAGATTTGCC	22	82
	R: GGTGTGACATTACTCCAGAGTTG	23	
Human LOX	F: CGGCGGAGGAAAACTGTCT	19	128
	R: TCGGCTGGGTAAGAAATCTGA	21	
Human SDF-1	F: ATTCTCAACACTCCAAACTGTGC	23	88
	R: ACTTTAGCTTCGGGTCAATGC	21	
Human TGF-β1	F: CAATTCCTGGCGATACCTCAG	21	86
	R: GCACAACTCCGGTGACATCAA	21	
Human GAPDH	F: ACAACTTTGGTATCGTGGAAGG	22	101
	R: GCCATCACGCCACAGTTTC	19	

Supplemental Figure: S1

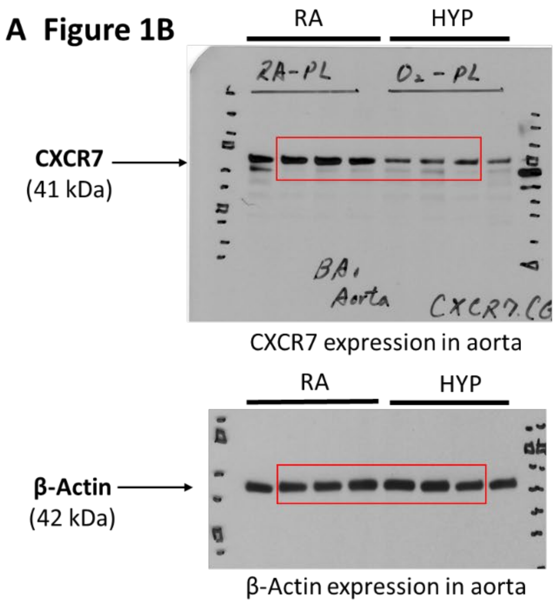

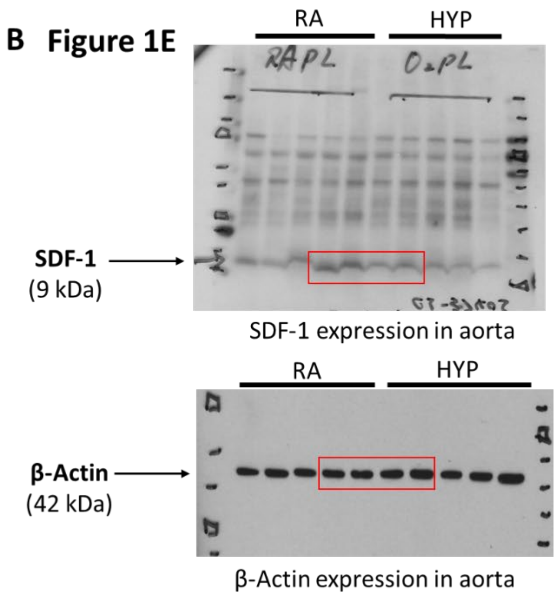

Supplemental figure S1 A-B: Original immunoblots for **(A)** Figure 1B, **(B)** Figure 1E of the manuscript. The full length immunoblots for β-Actin for Figures 1B and 1E could not be provided because the blots were cut prior to hybridization with antibody.

Supplemental Figure: S1

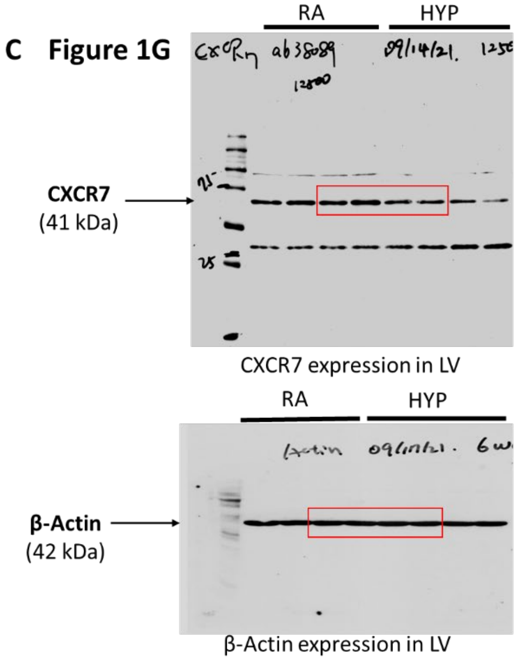

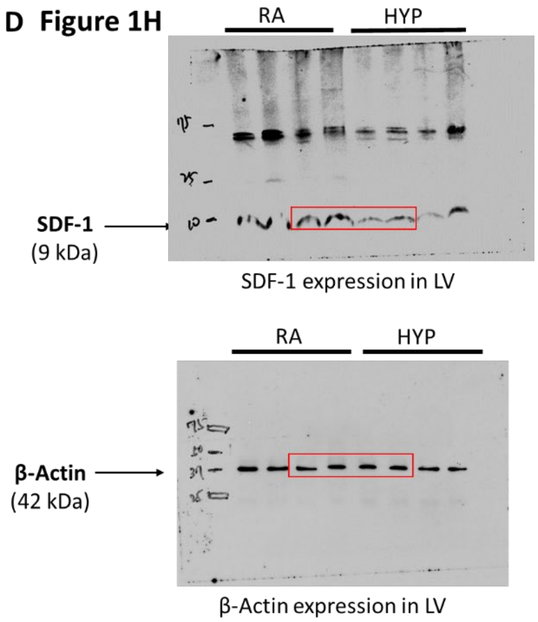

Supplemental figure S1 C-D: Original immunoblots for **(C)** Figure 1G, **(D)** Figure 1H of the manuscript. Red boxes represent part of the blots represented in the manuscript. RA=room air; HYP=hyperoxia.

Supplemental Figure: S1

E Figure 4A

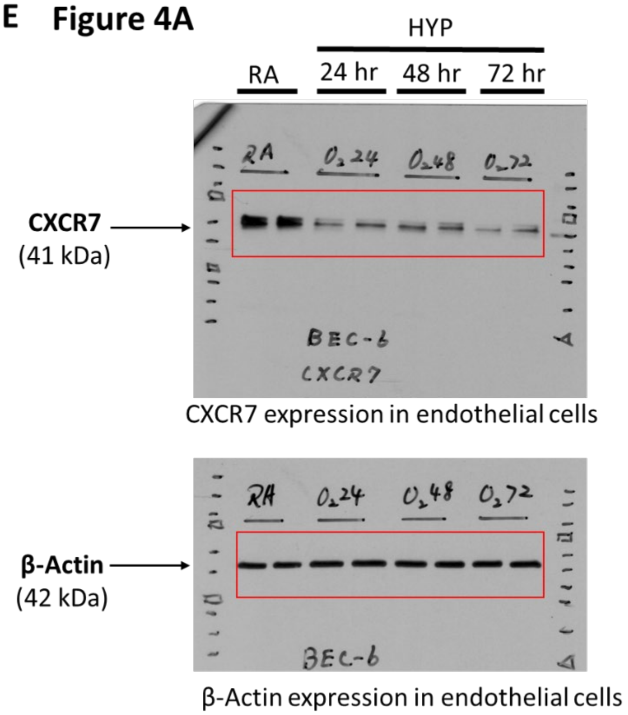

Supplemental figure S1 E: Original immunoblots for **(E)** Figure 4A of the manuscript. The full length immunoblot for β-Actin for Figure 4A could not be provided because the blot was cut prior to hybridization with antibody. Red boxes represent part of the blots represented in the manuscript. RA=room air; HYP=hyperoxia.

Supplemental Figure: S2

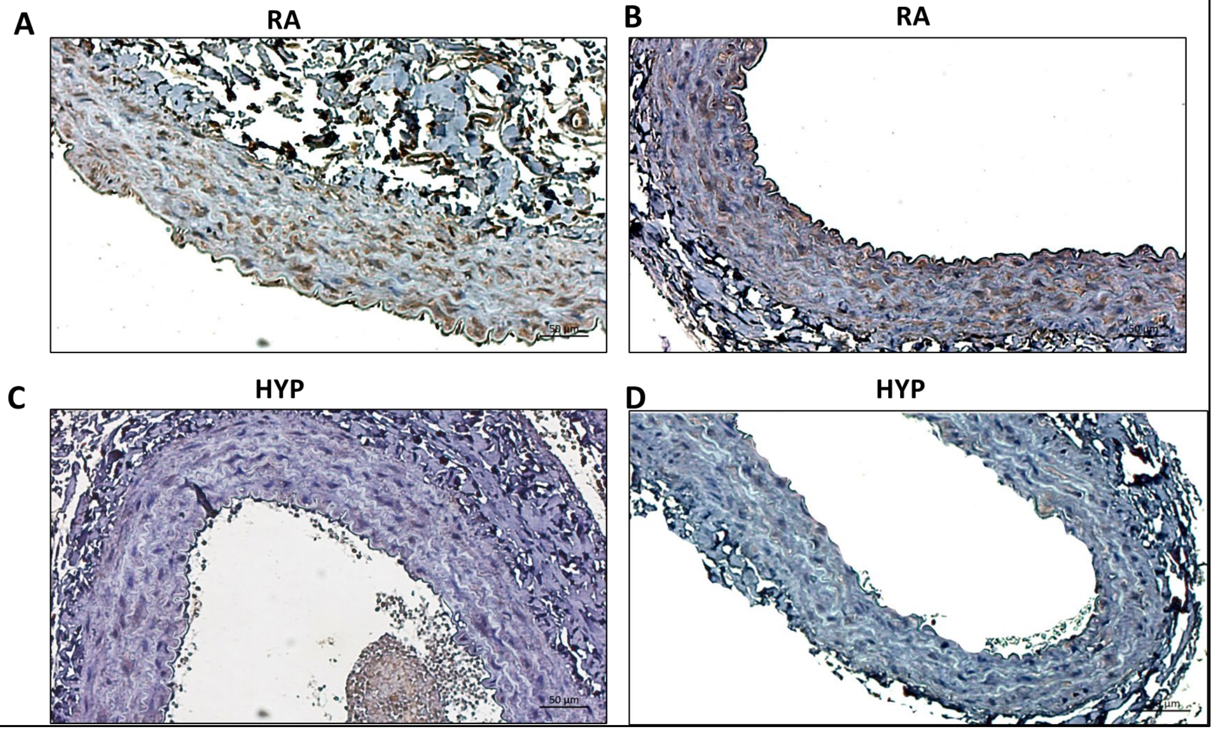

Supplemental Figure: S2

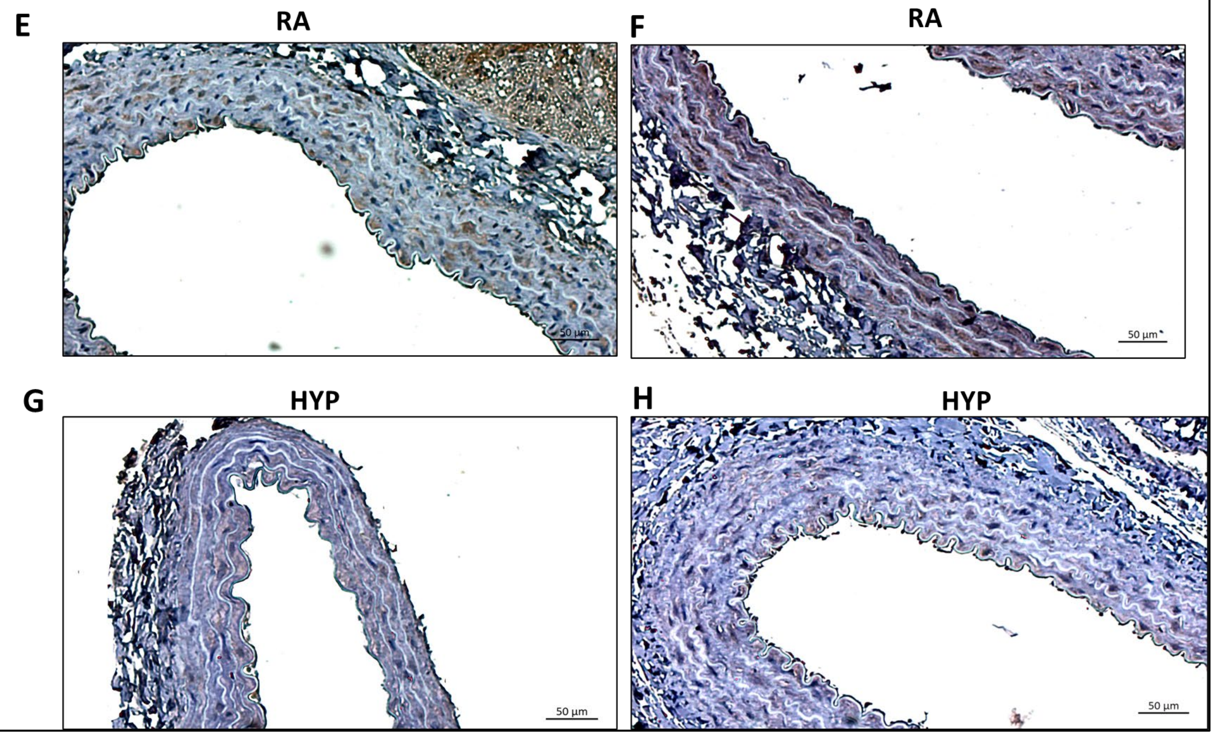

Supplemental Figure: S2

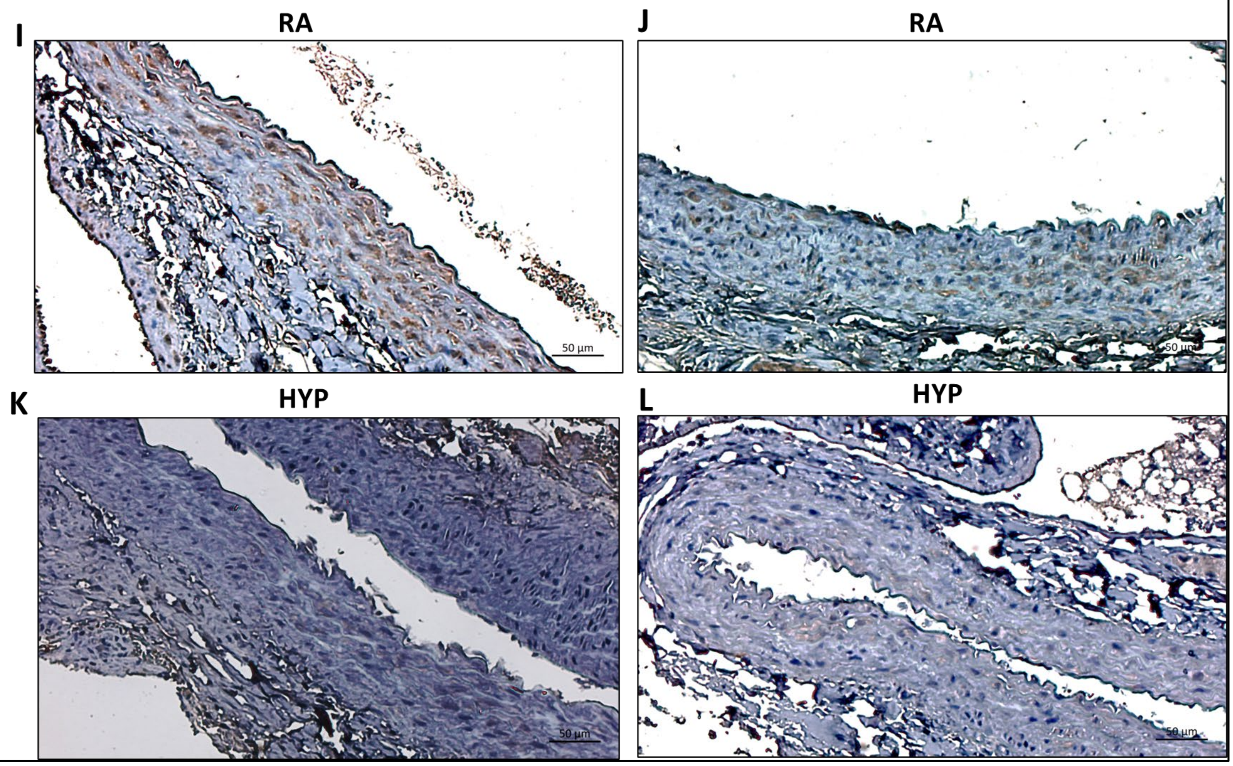

Supplemental figure S2: Representative immunohistochemical staining for CXCR7 in the aortic wall showing CXCR7 expression in normoxia (upper panel- Figures **A, B, E, F, I, J**) and hyperoxia-exposed aortas (lower panel- Figures **C, D, G, H, K, L**) (scale bars=50µm; n=6-7 per group). RA=room air; HYP=hyperoxia.
